# Risk of new onset autoimmune disease in 9- to 25-year-old women exposed to human papillomavirus-16/18 AS04-adjuvanted vaccine in the United Kingdom

**DOI:** 10.1080/21645515.2016.1199308

**Published:** 2016-07-18

**Authors:** Corinne Willame, Dominique Rosillon, Julia Zima, Maria-Genalin Angelo, Anke L. Stuurman, Hilde Vroling, Rachael Boggon, Eveline M. Bunge, Manel Pladevall-Vila, Laurence Baril

**Affiliations:** a Business & Decision Life Sciences on behalf of GSK Vaccines, Wavre, Belgium; b GSK Vaccines, Wavre, Belgium; c Pallas Health Research and Consultancy, Rotterdam, the Netherlands; d CPRD Research Group, Victoria, London, UK; e RTI Health Solutions, Barcelona, Spain; f Center for Health Policy and Health Services Research, Henry Ford Health System, Detroit, MI, USA

**Keywords:** autoimmune conditions, human papilloma virus, human papillomavirus vaccine, post-licensure safety study, vaccination, vaccine safety

## Abstract

To assess the risk of autoimmune disease (AD) in 9–25 year-old women within 1 year after the first AS04-HPV-16/18vaccine dose, a retrospective, observational database cohort study was conducted using CPRD GOLD. From CPRD GOLD 4 cohorts (65,000 subjects each) were retrieved: 1 exposed female cohort (received ≥1 AS04-HPV-16/18 vaccine dose between Sep2008–Aug2010) and 3 unexposed cohorts: historical female (Sep2005–Aug2007), concurrent male, and historical male. Co-primary endpoints were confirmed neuroinflammatory/ophthalmic AD and other AD, secondary endpoints were confirmed individual AD. Risk of new onset of AD was compared between cohorts (reference: historical cohort) using Poisson regression. The main analysis using confirmed cases showed no neuroinflammatory/ophthalmic AD cases in the female exposed cohort. Incidence rate ratio (IRR) (95% CI) of other AD was 1.41 (0.86 to 2.31) in female and 1.77 (0.94 to 3.35) in male cohorts when compared to the female and male historical cohort, respectively. Secondary endpoints were evaluated for diseases with >10 cases, which were Crohn's disease (IRR: 1.21 [0.37 to 3.95] for female and 4.22 [0.47 to 38.02] for male cohorts), autoimmune thyroiditis (IRR: 3.75 [1.25 to 11.31] for female and no confirmed cases for male cohorts) and type 1 diabetes (IRR: 0.30 [0.11 to 0.83] for female and 2.46 [1.08 to 5.60] for male cohorts). Analysis using confirmed and non-confirmed cases showed similar results, except for autoimmune thyroiditis in females, IRR: 1.45 (0.79 to 2.64). There was no evidence of an increased risk of AD in women aged 9 to 25 years after AS04-HPV-16/18 vaccination.

## Introduction

Human papillomavirus (HPV) is the main cause of cervical cancer,^1^ of which approximately 70% is caused by types 16 and 18.^2^ *Cervarix*^TM^ (AS04-HPV-16/18 vaccine) is a GSK Vaccines' bivalent recombinant vaccine against HPV types 16 and 18. Efficacy and cross-protective efficacy of this AS04-HPV-16/18 vaccine against persistent infection, pre-cancerous lesions, and cervical cancers caused by oncogenic HPV was shown in the Papilloma Trial against Cancer In young Adults (PATRICIA) and, more recently in adult women from the Human Papilloma Virus: Vaccine Immunogenicity And Efficacy (VIVIANE) study.^3-5^

Generally, pre-licensure clinical studies provide key vaccine safety data, but their power to detect rare events such as new onset of autoimmune diseases (AD) is limited by their sample size, since incidence rates of different AD vary roughly from 1 to 50 per 100,000 person-years.^6^

The use of appropriate adjuvants can help to modulate optimally innate and adaptive immune responses following vaccination. However, the risk of developing an autoimmune response provoked by the adjuvant itself cannot be ruled out.^7^

The Center for Biologics Evaluation and Research in the United States (US) requested GSK to conduct a post-licensure study to investigate the risk of AD among AS04-HPV-16/18 vaccine recipients. A pooled safety analysis of data from 57,580 adolescent and adult females aged 9 years and above, of whom 33,339 received at least one dose of AS04-HPV-16/18vaccine, showed that the rates of adverse events, medically significant conditions, serious adverse events, and potential immune-mediated disorders were similar between HPV and control groups.^8^

The aim of this observational cohort study using the Clinical Practice Research Datalink General Practice OnLine Database (CPRD GOLD) was to evaluate the risk of new onset of AD in women aged 9 to 25 years in the United Kingdom (UK) after administration of the AS04-HPV-16/18vaccine (exposed cohort) and in controls of the same age (unexposed cohorts).

## Results

From a total of 168,662 HPV vaccinated female subjects in CPRD, 103,081 (61.12%) were eligible for the exposed cohort. The number of eligible subjects in the other cohorts was: 107,434 for the unexposed historical female cohort, 142,772 for the concurrent male cohort and 92,337 for the historical male cohort. 65,000 Subjects were randomly selected from each of the cohorts but 42 subjects were excluded because a de-enrolment date (death date or date of lost to follow-up) occurred before the study start date.

**Figure 1.**
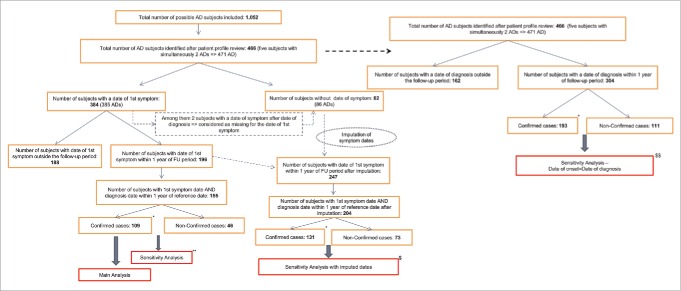
Number of cases included in each analysis. AD = Autoimmune Disease; FU = follow-up. *Confirmation of cases was performed after subject profile review. **The 46 non-confirmed cases were combined with the 109 confirmed cases in the sensitivity analysis for subjects with known first symptom dates. ^$^Subjects for the imputed dates sensitivity analyses had either an imputed date of first symptom or a known date of first symptom. Sensitivity analyses for subjects with imputed/known first symptom dates were repeated using either confirmed cases only or confirmed and non-confirmed cases. ^$$^ Date of onset was assumed to be the same as date of disease diagnosis in this sensitivity analysis. Sensitivity analyses were repeated using either confirmed cases only or confirmed and non-confirmed cases.

Through the pre-defined algorithms 1,052 suspected AD cases were identified, of which 466 (44.3%) were identified as having confirmed or non-confirmed new onset AD after review of the individual subject profiles (Fig. 1). Among them, the date of first symptom was known for 384 (82.4%) cases, of which 40.4% (n = 155) were eligible for the main analysis, because their first symptom date and date of disease diagnosis were within the one year follow-up period. Out of these 155 AD cases, 109 (70.3%) were classified as confirmed cases and were included in the numerator in the main analysis (the 46 non-confirmed cases were excluded from the numerator and their person-time was included in the denominator). The number of cases included during sensitivity analyses can be found in Figure 1. A total of 68 confirmed cases from the exposed cohort were included for the self-controlled case-series (SCCS) analysis.

The overall population for the main analysis contained 259,876 subjects. Demographic and baseline characteristics of each cohort are depicted in Table 1. The female exposed cohort more frequently used healthcare, had more years of follow-up in CPRD GOLD at the study start date, and received more vaccines during the follow-up period than the historical female cohort. Similar differences existed between the 2 male cohorts, except that the male concurrent cohort received fewer vaccines in the year prior to the study start date than the male historical cohort.

**Table 1.** Demographic characteristics of the 4 cohorts.

		AS04-HPV-16/18 vaccine exposure N = 64,964		Unexposed historical female cohort N = 64,973		Unexposed concurrent male cohort N = 64,974		Unexposed historical male cohort N = 64,965	
Characteristics	Parameters or categories	Value or n	%	Value or n	%	Value or n	%	Value or n	%
Age at study start date (years)	Mean (SD)	15.3 (2.1)	—	15.4 (2.1)	—	15.3 (2.1)	—	16.0 (2.0)	—
	Range	9.4–24.9	—	9.4–24.8	—	9.3–24.9	—	9.2–24.8	–
	9–13	20,654	31.8	19,783	30.4	21,252	32.7	13,361	20.6
	14–17	38,082	58.6	38,872	59.8	37,990	58.5	42,871	66.0
	18–21	6,199	9.5	6,291	9.7	5,708	8.8	8,689	13.4
	22–25	29	<0.1	27	<0.1	24	<0.1	44	<0.1
Region of GP practices	North England	36,818	56.7	34,646	53.3	35,906	55.3	33,247	51.2
	Midlands	8,396	12.9	8,556	13.2	8,423	13.0	8,724	13.4
	South England	19,648	30.2	21,733	33.4	20,616	31.7	22,971	35.4
	Ireland Scotland Wales	102	0.2	38	<0.1	29	<0.1	23	<0.1
Available HES linkage	Yes	38,656	59.5	36,148	55.6	37,832	58.2	37,616	57.9
Number of healthcare resources utilization^*^ the year prior to the study start date	Mean (SD)	8.8 (10.2)	—	7.0 (9.1)	—	6.0 (8.4)	—	5.3 (7.2)	—
	Range	0–243	—	0–157	—	0–254	—	0–132	—
	0 to 1	12,203	18.8	17,940	27.6	21,057	32.4	22,445	34.5
	2 to 4	15,746	24.2	17,056	26.3	17,448	26.9	18,262	28.1
	5 to 9	16,113	24.8	14,454	22.2	13,362	20.6	13,186	20.3
	≥10	20,902	32.2	15,523	23.9	13,107	20.2	11,072	17.0
Number of years of follow-up in CPRD GOLD at study start date	Mean (SD)	9.4 (4.3)	—	7.6 (4.3)	—	9.1 (4.3)	—	7.8 (4.4)	—
	Range	1–21	—	1–19	—	1–21	—	1–19	—
	0 to 3	5,646	8.7	9,497	14.6	7,062	10.9	9,486	14.6
	3 to 6	9,638	14.8	16,923	26.0	10,054	15.5	16,510	25.4
	6 to 10	20,456	31.5	20,927	32.2	20,581	31.7	20,924	32.2
	≥10	29,224	45.0	17,626	27.1	27,277	42.0	18,045	27.8
Exposure to vaccines in the year prior to the study start date	Any vaccine	11,529	17.7	11,008	16.9	9,270	14.3	10,394	16.0
	Novel adjuvanted vaccine	311	0.5	0	0.0	325	0.5	0	0.0
	Live-attenuated vaccine	1,138	1.8	2,986	4.6	861	1.3	2,942	4.5
	Other vaccine	10,627	16.4	8,580	13.2	8,507	13.1	7,967	12.3
Exposure to vaccines in the follow-up period	Any vaccine	11,596	17.8	7,765	12.0	8,000	12.3	6,253	9.6
	Novel adjuvanted vaccine	1,679	2.6	0	0.0	1,559	2.4	0	0.0
	Live-attenuated vaccine	1,033	1.6	943	1.5	489	0.8	828	1.3
	Other vaccine	10,231	15.7	7,062	10.9	7,068	10.9	5,578	8.6

*Note.* HES = Hospital Episode Statistics; N = number of subjects; n/% = number/percentage of subjects in a given category; SD = standard deviation; Value = value of the considered parameter.

* E.g. general practioner consultations, prescriptions, and laboratory tests.

### Co-primary endpoints

In total, 3 confirmed cases of neuroinflammatory/ophthalmic AD and 106 confirmed cases of other AD were observed within the one year follow-up period (Table 2). There were no confirmed cases of neuroinflammatory/ophthalmic AD in the exposed female cohort, therefore the incidence rate ratio (IRR) could not be calculated. The corresponding age-adjusted IRR for male cohorts was 0.95 (95% confidence interval (CI): 0.06–15.18). For the other AD, the age-adjusted IRRs were 1.41 (95% CI: 0.86–2.31) for the female cohorts and 1.77 (95% CI: 0.94–3.35) for the male cohorts.

**Table 2.** Incidence rate per 100,000 person-years and incidence rate ratios^*^ of co-primary endpoints.

		AS04-HPV-16/18 vaccine exposure (total PY = 64,705)		Unexposed historical female cohort (total PY = 64,841)		
Diseases		n	IR per 100,000 PY (95% CI)	n	IR per 100,000 PY (95% CI)	IRR^*^ (95% CI) EXP/NNEXP
Co-primary endpoints						
Neuroinflammatory/ophthalmic AD	Confirmed cases	0	0.00 (0.00–5.70)	1	1.54 (0.04–8.59)	—
	All cases	4	6.18 (1.68–15.83)	7	10.80 (4.34–22.24)	0.57 (0.17–1.96)
Other AD	Confirmed cases	38	58.73 (51.56–80.61)	27	41.64 (27.44–60.58)	1.41 (0.86–2.31)
	All cases	51	78.82 (58.69–103.63)	41	63.23 (45.38–85.78)	1.25 (0.83–1.88)
		**Unexposed concurrent male cohort (total PY = 64,859)**		**Unexposed historical male cohort (total PY = 64,868)**		
**Diseases**		**n**	**IR per 100,000 PY (95% CI)**	**n**	**IR per 100,000 PY (95% CI)**	IRR* **(95% CI) MALE/HIST**
**Co-primary endpoints**						
Neuroinflammatory/ophthalmic AD	Confirmed cases	1	1.54 (0.04–8.59)	1	1.54 (0.04–8.59)	0.95 (0.06–15.18)
	All cases	3	4.63 (0.95–13.52)	2	3.08 (0.37–11.14)	1.73 (0.29–10.47)
Other AD	Confirmed cases	26	40.09 (26.19–58.74)	15	23.12 (12.94–38.14)	1.77 (0.94–3.35)
	All cases	28	43.17 (28.69–62.39)	19	29.29 (17.64–45.74)	1.52 (0.85–2.73)

*Note.* AD = autoimmune disease; CI = confidence interval; EXP = AS04-HPV-16/18 vaccine exposure; HIST = unexposed historical male cohort; IRR = incidence rate ratio; MALE = unexposed concurrent male cohort; n = number of subjects; NNEXP = unexposed historical female cohort; PY = person-years

* Adjusted for age group (9–17 years, 18–25 years)

Sensitivity analysis using confirmed and non-confirmed cases showed results similar to the main analysis (Table 2), as all the sensitivity analyses using the 2 other case definitions (Tables S1 and S2); models including more covariates (data not shown); and the SCCS analysis (Table S3).

### Individual diseases with >10 cases in female cohorts

Table 3 gives the number of cases per individual disease and the corresponding incidence rate in each of the 4 cohorts. There were 3 diseases for which more than 10 cases were found in the female cohorts, namely autoimmune thyroiditis, Crohn's disease, and type 1 diabetes. For autoimmune thyroiditis a significant increased risk was found in the female exposed cohort (IRR 3.75, 95% CI: 1.25–11.31) (Table 4). No IRR for males could be calculated as no confirmed cases were found in either male cohort. The IRR for Crohn's disease was 1.21 (95% CI: 0.37–3.95) for females and 4.22 (95% CI: 0.47–38.02) for males. For type 1 diabetes, the IRR was 0.50 (95%CI: 0.21–1.17) for females, while a significant increased risk was found in the concurrent male cohort (IRR 2.46, 95% CI: 1.08–5.60). A significant decreased risk of type 1 diabetes was found in the female exposed cohort, when adjusted for male effect (IRR 0.30, 95% CI: 0.11–0.83).

**Table 3.** Incidence rate per 100,000 person-years of individual autoimmune diseases.

		AS04-HPV-16/18 vaccine exposure (total PY = 64,705)		Unexposed historical female cohort (total PY = 64,841)		Unexposed concurrent male cohort (total PY = 64,859)		Unexposed historical male cohort (total PY = 64,868)	
Diseases		n	IR per 100,000 PY	n	IR per 100,000 PY	n	IR per 100,000 PY	n	IR per 100,000 PY
Acute disseminated encephalomyelitis	Confirmed cases	0	0.00	0	0.00	0	0.00	0	0.00
	All cases	1	1.55	0	0.00	0	0.00	0	0.00
Autoimmune thyroiditis	Confirmed cases	15	23.18	4	6.17	0	0.00	0	0.00
	All cases	26	40.18	18	27.76	2	3.08	3	4.63
Autoimmune uveitis	Confirmed cases	0	0.00	0	0.00	0	0.00	0	0.00
	All cases	2	3.09	5	7.71	2	3.08	1	1.54
Crohn's disease	Confirmed cases	6	9.27	5	7.71	4	6.17	1	1.54
	All cases	8	12.36	5	7.71	4	6.17	2	3.08
Guillain-Barré syndrome	Confirmed cases	0	0.00	0	0.00	1	1.54	1	1.54
	All cases	0	0.00	0	0.00	1	1.54	1	1.54
Idiopathic thrombo-cytopenic purpura	Confirmed cases	1	1.55	1	1.54	0	0.00	2	3.08
	All cases	1	1.55	1	1.54	0	0.00	2	3.08
Inflammatory bowel disease	Confirmed cases	0	0.00	0	0.00	0	0.00	1	1.54
	All cases	0	0.00	0	0.00	0	0.00	1	1.54
Juvenile rheumatoid arthritis	Confirmed cases	1	1.55	0	0.00	0	0.00	1	1.54
	All cases	1	1.55	0	0.00	0	0.00	1	1.54
Multiple sclerosis	Confirmed cases	0	0.00	1	1.54	0	0.00	0	0.00
	All cases	0	0.00	1	1.54	0	0.00	0	0.00
Optic neuritis	Confirmed cases	0	0.00	0	0.00	0	0.00	0	0.00
	All cases	1	1.55	1	1.54	0	0.00	0	0.00
Other AD^*^	Confirmed cases	1	1.55	0	0.00	0	0.00	0	0.00
	All cases	1	1.55	0	0.00	0	0.00	0	0.00
Psoriatic arthritis	Confirmed cases	1	1.55	1	1.54	0	0.00	0	0.00
	All cases	1	1.55	1	1.54	0	0.00	0	0.00
Rheumatoid arthritis	Confirmed cases	1	1.55	0	0.00	0	0.00	0	0.00
	All cases	1	1.55	0	0.00	0	0.00	0	0.00
Type 1 diabetes mellitus	Confirmed cases	8	12.36	16	24.68	20	30.84	8	12.33
	All cases	8	12.36	16	24.68	20	30.84	8	12.33
Ulcerative colitis	Confirmed cases	4	6.18	0	0.00	2	3.08	2	3.08
	All cases	4	6.18	1	1.54	2	3.08	2	3.08

*Note.* n = number of subjects; PY = person-years

* AD = autoimmune disease, includes acute disseminated encephalomyelitis and autoimmune peripheral neuropathies and plexopathies

**Table 4.** Incidence rate per 100,000 person-years and incidence rate ratios^*^ of individual autoimmune diseases with >10 cases in female cohorts.

		AS04-HPV-16/18 vaccine exposure (total PY = 64,705)		Unexposed historical female cohort (total PY = 64,841)		
Diseases		n	IR per 100,000 PY (95% CI)	n	IR per 100,000 PY (95% CI)	IRR^*^ (95% CI) EXP/NNEXP
Autoimmune thyroiditis	Confirmed cases	15	23.18 (12.98;38.24)	4	6.17 (1.68; 15.80)	3.75 (1.25–11.31)
	All cases	26	40.18 (26.25;58.88)	18	27.76 (16.45;43.87)	1.45 (0.79–2.64)
Crohn's disease	Confirmed cases	6	9.27 (3.40;20.18)	5	7.71 (2.50;18.00)	1.21 (0.37–3.95)
	All cases	8	12.36 (5.34;24.26)	5	7.71 (2.50;18.00)	1.61 (0.53–4.91)
Type 1 diabetes mellitus	Confirmed cases	8	12.36 (5.34;24.36)	16	24.68 (14.10;40.07)	0.30 (0.11–0.83)^**^
	All cases	8	12.36 (5.34;24.36)	16	24.68 (14.10;40.07)	0.50 (0.21–1.17)
		**Unexposed concurrent male cohort (total PY = 64,859)**		**Unexposed historical male cohort (total PY = 64,868)**		
**Diseases**		**n**	**IR per 100,000 PY (95% CI)**	**n**	**IR per 100,000 PY (95% CI)**	IRR^*^(95% CI) MALE/HIST
Autoimmune thyroiditis	Confirmed cases	0	0.00 (0.00; 5.69)	0	0.00 (0.00; 5.69)	—
	All cases	2	3.08 (0.37;11.14)	3	4.63 (0.95;13.52)	0.76 (0.13–4.60)
Crohn's disease	Confirmed cases	4	6.17 (1.68;15.79)	1	1.54 (0.04;8.59)	4.22 (0.47–38.02)
	All cases	4	6.17 (1.68;15.79)	2	3.08 (0.37;11.14)	2.06 (0.38–11.34)
Type 1 diabetes mellitus	Confirmed cases	20	30.84 (18.84;47.62)	8	12.33 (5.32;24.30)	2.46 (1.08–5.60)
	All cases	20	30.84 (18.84;47.62)	8	12.33 (5.32;24.30)	2.46 (1.08–5.60)

*Note.* CI = confidence interval; EXP = AS04-HPV-16/18 vaccine exposure; HIST = unexposed historical male cohort; IRR = incidence rate ratio; MALE = unexposed concurrent male cohort; n = number of subjects; NNEXP = unexposed historical female cohort; PY = person-years

* Adjusted for age group (9–17 years, 18–25 years)

** The IRR for confirmed type 1 diabetes in the female cohorts was adjusted for the male effect, because a significant difference in incidence rates was observed between the 2 male cohorts

Sensitivity analysis using confirmed and non-confirmed cases showed similar results, except for autoimmune thyroiditis in females, for which a lower and non-significant IRR of 1.45 (95% CI: 0.79–2.64) was found (Table 4). No significant IRRs were found for females and males in any of the other sensitivity analyses (2 other case definitions: Tables S1 and S2, models using other covariates: data not shown), or in the SCSS analysis (Table S3).

### Post-hoc analyses for the autoimmune thyroiditis cases

The number of autoimmune thyroiditis cases appeared to decrease over time during the one year follow-up period in all cohorts. This was also seen for the other AD.

After additional medical record review, most of the 49 autoimmune thyroiditis cases were classified as hypothyroiditis (81.6%). Corresponding IRRs for autoimmune hypothyroiditis in the female cohorts were 3.00 (95% CI: 0.97–9.31) for confirmed cases and 1.47 (95% CI: 0.76–2.83) for confirmed and non-confirmed cases. No confirmed autoimmune hypothyroiditis cases were found in the male cohorts, but when considering confirmed and non-confirmed cases the IRR was 1.90 (95% CI: 0.17–20.94). These results confirm the estimates in females for all autoimmune thyroiditis (hypothyroiditis and hyperthyroiditis combined).

After exclusion of subjects from the Northern Ireland, Scotland, and Wales regions (as per post-hoc analysis), a non-significant IRR for confirmed autoimmune thyroiditis was found (IRR 2.50, 95% CI: 0.79–7.98).

### Discussion

The main analysis based on confirmed cases showed no significant IRRs for any of the co-primary endpoints. However, among the most frequent AD for which symptom start dates are difficult to establish, the risk of autoimmune thyroiditis was increased and the risk of type 1 diabetes was decreased in the female vaccinated cohort. Sensitivity analysis using all cases (i.e. confirmed and non-confirmed) showed similar results, except for autoimmune thyroiditis in which the IRR was not significant anymore. The findings on autoimmune thyroiditis and type 1 diabetes from the main analysis were not confirmed in sensitivity analyses using other case definitions, nor in the SCCS analysis.

A publication by the UK Medicines and Healthcare products Regulatory Agency (MHRA) reviewed the safety profile of AS04-HPV-16/18vaccine use in the UK from September 2008 to July 2012, when over 6 million doses of the vaccine had been given across the UK, and identified no new safety concerns.^9^ Randomized clinical studies did not show any increased risk of AD in the vaccinated group compared to controls.^8,10,11^ A post-licensure safety surveillance of routine use of AS04-HPV-16/18vaccine did not find any pattern or trend for potential immune-mediated diseases after vaccination.^12^ This current vaccine post-licensure study confirms the overall acceptable safety profile of AS04-HPV-16/18vaccine.

Research by Chao, in which the Kaiser Permanente Database was used and AD cases were found using similar case identification and ascertainment methods, showed an increased risk of Grave's and Hashimoto diseases combined and a decreased risk of type 1 diabetes after 4 vHPV vaccination.^13^ A study by Arnheim-Dahlström using healthcare registers from Denmark and Sweden, on the contrary, found an increased risk of type 1 diabetes after 4 vHPV vaccination.^14^ However, both authors concluded that there was no clear evidence of a safety signal following vaccination with 4vHPV, because no cluster of disease onset in relation to vaccination timing was found and no significant increased risk of most other conditions was found in vaccinated women. Moreover, in a follow-up review of the study by Chao, the authors concluded that many of the confirmed incident Grave's disease cases were actually prevalent cases.^15^ A recent observational study carried out in a cohort of approximately 4 million women aged 10 to 44 years in Denmark did not find an increased risk of multiple sclerosis or other demyelinating diseases after 4 vHPV vaccination.^16^ Additionally, other observational studies did not find any increased risk of AD in the 4 vHPV vaccinated group compared to an unvaccinated group.^17-19^

The incidence of autoimmune thyroiditis in the vaccinated cohort was within the same range as the one in CPRD GOLD for the studied age group (incidence rates from the feasibility assessment for the period 2008–2010: age group [9–18] = 1.22 and 5.52/100,000 person-years respectively in males and females, age group [18–25] = 1.88 and 8.30/100,000 person-years respectively in males and females), indicating that although we found a significantly increased incidence in the exposed cohort, this was still within expected ranges. The increased incidence of autoimmune thyroiditis could be explained by a change in diagnostic methods over time.

Our study had a number of limitations. First, CPRD GOLD is based on data from general practices (GP), while most autoimmune diseases are probably diagnosed in specialist settings. Not all GPs participating in CPRD GOLD consented to the linkage between CPRD GOLD primary care data and Hospital Episodes Statistics (HES) data (linkage was around 50% as of the first quarter of 2013). Consequently, the number of autoimmune diseases, the quality of the information, and the diagnostic certainty might be limited. In particular, the specific information related to the onset of clinical symptoms, and radiological, biological and genetic predisposition data associated with the etiologic diagnosis of AD may not have all been available in the CPRD GOLD database and associated resources. This is reflected in the low confirmation rate for some of the AD (i.e., autoimmune thyroiditis, autoimmune uveitis). Second, when the first symptom of an AD for a subject was known but the date of onset of the symptom was not known (i.e. there was no indication regarding the date on which the first symptom started), the date of first report of this symptom was used as date of first symptom. This is a limitation in the main analysis and sensitivity analysis using both known and imputed dates of first symptom, because it is highly likely that in a subset of these subjects the symptom has started (much) earlier, possibly before the first dose of AS04-HPV-16/18vaccine. Third, analyses for the first co-primary endpoint (neuroinflammatory/ophthalmic AD) and most of the individual AD were not possible due to the small number of cases. Fourth, an additional limitation could be the risk of identifying false negative cases (lack of sensitivity). The case ascertainment procedure ensured a high specificity of the endpoint(s), but the team did not review the subject profiles of the non-cases (because unfeasible for 65,000 subjects per cohort), and this means that possible cases of ADs could have been missed. However a high specificity was required to avoid a bias toward the null hypothesis whereas high sensitivity was not essential. Lack of sensitivity does not bias the risk estimate, but could impact the precision resulting in a somewhat broader confidence interval. Fifth, the number of AD cases seemed to decrease over time during the one year follow-up period in all 4 cohorts. This could potentially be explained by our study design: a diagnosis of AD was searched in the database through algorithms during the one year period after the first AS04-HPV-16/18vaccine dose or equivalent study start date and then it was verified by medical review whether the onset of symptoms occurred during this period. It is plausible that cases of AD with onset of symptoms late during the one year follow-up period were not detected because the diagnosis was reported later than one year after the study start date. However, we feel that only a few cases might have been missed as the onset of several AD is (sub)acute. Sixth, studies of rare events typically have low power and therefore only large risk increases can be detected. The present study shares this limitation. To overcome this, 2 composite co-primary endpoints were defined. The observed incidence of the co-primary endpoint ‘other autoimmune diseases’ was in alignment with the sample size calculation assumptions, but it was lower than expected for the neuroinflammatory diseases. However, the absence of confirmed neuroinflammatory disease cases in the exposed cohort was quite re-assuring. Lastly, multiple endpoint comparisons increase the overall type I error. However, no adjustment for type I error is also the most conservative approach for safety endpoints since it avoids masking possible signals.

Despite these limitations, we still think this study performed well. A major strength of this study was that it was based on a large population-based database that is likely to be representative of young women and men in the UK. The use of the CPRD GOLD database provided a unique opportunity to study the effect of AS04-HPV-16/18vaccine on the occurrence of AD as AS04-HPV-16/18vaccine was used during 3 years in a universal mass vaccination program for young women in the UK. New onset of AD was assessed by thorough subject data review, combining data from CPRD, HES and free text, and using several case ascertainment steps including expert review. This procedure provides a high specificity of the endpoints which is crucial to minimize the risk of bias to the null hypothesis. Attempts were made to minimize case ascertainment bias by blinding experts for HPV vaccine status during case review. In order to prevent inclusion of vaccinated subjects in an ‘unexposed’ cohort, the vaccinated exposed cohort was compared to a historical unexposed cohort before the start of the AS04-HPV-16/18 vaccine program in the UK. In addition, 2 unexposed male cohorts were enrolled in order to assess a possible change over time in reporting AD in CPRD GOLD independent of AS04-HPV-16/18vaccine introduction. Though incidence rates of autoimmune diseases differ across gender, the male cohorts were used as an internal control. Finally, for the exposed cohort, an additional SCCS analysis was performed in order to control for all fixed confounders not varying with time during the follow-up period. Age-stratified analyses were also performed and generated consistent results (not presented here).

### Conclusion

This observational study did not show evidence of an increased risk of AD following vaccination with AS04-HPV-16/18 vaccine. No significant IRRs were found for the co-primary endpoints in the female cohorts. However, a significant increased risk of autoimmune thyroiditis (IRR=3.75, 95% CI: 1.25–11.31) and a significant decreased risk of type 1 diabetes (after adjustment for male effect, IRR=0.30, 95% CI: 0.11–0.83), was found in the female cohorts using confirmed cases only. Using all cases (i.e., confirmed and non-confirmed) showed similar results, except for autoimmune thyroiditis in which the IRR was not significant anymore. Sensitivity analyses using other case definitions and the SCCS analysis did not find any significant IRR between the exposed and unexposed female cohorts.

## Materials and methods

### Data source, population and setting

CPRD GOLD is one of the largest anonymised primary care databases, and captures longitudinal medical records including clinical events, laboratory results, drug prescriptions, referrals to specialists, and immunisation records from over 680 GP's in the UK.^20^ Linkage between CPRD GOLD primary care data and HES data was available for approximately 50% of subjects as of the beginning of 2013.^21^ Complementary information to coded GP data can be obtained through the free text data captured in the practice management system from CPRD GOLD.^20^ Free text data include notes or documents entered or scanned in by the GP, including letters from specialists in secondary or private care settings.

A public immunization program targeting girls between 12–13 years of age including a catch-up program for young women up to 18 years was undertaken in the UK during the academic year 2008/09. The phased catch-up program for females born 1 September 1991 to 31 August 1995 during the 2008/09 academic year was completed by the end of the 2009/10 academic year. The program was delivered largely through secondary schools.^22-24^ In the UK public HPV immunization program (12–13 year olds), HPV vaccination coverage for 2010/11 was 89.0%, 87.6% and 83.8% for the first, second and third dose respectively.^25^ The bivalent vaccine was replaced in the program by the tetravalent vaccine *Gardasil* (4vHPV; Merck & Co) in September 2012.

**Figure 2.**
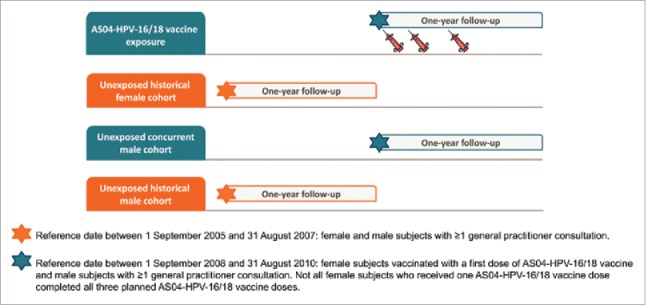
Cohort design. Reference date between 1 September 2005 and 31 August 2007: female and male subjects with ≥1 general practitioner consultation. Reference date between 1 September 2008 and 31 August 2010: female subjects vaccinated with a first dose of AS04-HPV-16/18 vaccine and male subjects with ≥1 general practitioner consultation. Not all female subjects who received one AS04-HPV-16/18 vaccine dose completed all 3 planned AS04-HPV-16/18 vaccine doses.

The study population included female and male subjects registered in CPRD GOLD for at least one year before the study start (Fig. 2). The female population was composed of subjects vaccinated with AS04-HPV-16/18 vaccine between the ages of 9 and 25 years and unexposed subjects of the same age identified from historical data. A historical unexposed cohort before the start of the *Cervarix* program in the UK was chosen in order to prevent inclusion of vaccinated subjects in an ‘unexposed’ cohort (because when no vaccination is reported in CPRD GOLD, it cannot be ruled out that the subject did receive the vaccine). The male population was composed of 9 to 25-year-old subjects not vaccinated with AS04-HPV-16/18 vaccine, comprising both a concurrent and a historical male cohort. Comparison of the unexposed concurrent male cohort with the unexposed historical male cohort was used as an internal control for changes over time in the incidence of AD in CPRDGOLD. Women who received at least one dose of AS04-HPV-16/18 vaccine administered according to local practice between 1 September 2008 and 31 August 2010 were eligible for the exposed group. Men with at least one GP consultation during the same period (concurrent male group), and women and men with at least one GP consultation between 1 September 2005 and 31 August 2007 (historical groups) were eligible for the unexposed groups. Subjects who received an unspecified HPV vaccine or 4 vHPV were excluded, as were unexposed subjects who received any dose of AS04-HPV16/18 vaccine at any time before the study period. Subjects with a diagnostic code of any AD during the year prior to the study start were also excluded.

The study protocol was approved by the Independent Scientific Advisory Committee for the MHRA database research. No patient informed consent was needed because patient information in CPRD GOLD is fully anonymised. The study is registered at www.clinicaltrials.gov (NCT01953822) and in the EU PAS Register (ENCEPP/SDPP/4584).

### Study cohorts

Four cohorts were defined based on exposure to AS04-HPV-16/18 vaccine and sex as recorded in the CPRD GOLD data source (Fig. 2): 1) female cohort vaccinated with AS04-HPV-16/18 vaccine (exposed), 2) unexposed historical female cohort, 3) unexposed concurrent male cohort, 4) unexposed historical male cohort. Subjects in the 3 unexposed cohorts were pre-selected after applying a frequency matching for age and practice region to the subjects included in the vaccinated (exposed) cohort. A random selection was applied to the pre-selected unexposed subjects in order to include the targeted number of subjects in each unexposed cohort. The study start date for the exposed cohort was the date of the first dose of AS04-HPV-16/18 vaccine. The study start date for the unexposed cohorts was a random date selected among the study start dates of the matched exposed cohort (minus 3 years for the historical cohorts).

### Outcome definition

The primary study outcome was the occurrence of new onset of 2 groups of confirmed AD during the period of one year following the study start date (follow-up period). These two co-primary composite endpoints have been defined as: 1) neuroinflammatory/ophthalmic diseases: multiple sclerosis, transverse myelitis, optic neuritis, Guillain-Barré syndrome, autoimmune uveitis, and other demyelinating diseases, or 2) other AD: systemic lupus erythematosus, rheumatoid arthritis, juvenile rheumatoid arthritis, Still's disease, psoriatic arthritis, ankylosing spondylitis, idiopathic thrombocytopenic purpura, autoimmune hemolytic anaemia, type 1 diabetes mellitus, autoimmune thyroiditis, Crohn's disease, ulcerative colitis, and autoimmune hepatitis.

Secondary outcomes included the occurrence of new onset of individual confirmed AD during the period of one year following the study start date (follow-up period).

The one-year follow-up period was chosen in agreement with the Food and Drug Administration (and the European Medicines Agency, and is supported by the article from Tavares *et al* on the optimal conduct of clinical trials of new vaccines investigating the risk of AD.^26^

### Data collection and case ascertainment

Subjects with suspected AD diagnoses were identified in CPRD GOLD and/or HES using pre-defined algorithms (the algorithm for Guillain-Barré syndrome is given in Tables S4 and S5 as an example, the other algorithms are available upon request). The final study database consisted of data for these subjects automatically extracted from CPRD GOLD (Tables S6 and S7), HES, and additional data from free text review. Information extracted included clinical diagnosis, laboratory testing, drug prescription, and HES-linked data. Specific de-identified free text associated with possible first symptoms, laboratory tests, drug prescriptions, and diagnosis of AD was requested when necessary in order to classify each subject as a confirmed new onset AD case, a non-confirmed new onset AD case, or a non-case. If a date of diagnosis did not fall within the follow-up period, a subject could not qualify as a case in any of the analyses. All subject profiles and requested free text were reviewed by Pallas, Health Research and Consultancy B.V., the Netherlands.

A safety physician from GSK and an external physician from Research Triangle Institute (RTI) Health Solutions reviewed all subjects with a doubtful outcome. Final case ascertainment was adjudicated by 5 independent external experts in the fields of rheumatology, ophthalmology, neurology, and internal medicine who remained blinded with respect to the exposure status of the subjects throughout the ascertainment process. Each expert reviewed the subjects, which included subjects with a doubtful outcome after review by Pallas, the GSK safety physician, and the RTI physician, and a 10% random sample of the remaining subjects per AD, according to their specialty. Fifty subjects were reviewed by the experts as part of the random check. Agreement on the date of first symptom, type of AD, confirmation of AD, and date of diagnosis existed for all subjects with rheumatology and neurology diagnoses and for most of the subjects with ophthalmology and internal medicine diagnoses. For autoimmune uveitis, however, the expert decided to include an additional first symptom (i.e. conjunctivitis/episcleritis) that had not been used by Pallas, GSK, and RTI. For inflammatory bowel disease, Crohn's diseases, and ulcerative colitis, the expert suggested other criteria to determine the date of diagnosis and confirmation of the diagnosis. All uveitis, inflammatory bowel disease, Crohn's disease, and ulcerative colitis subjects were therefore reviewed again by Pallas applying the revised criteria. Furthermore, after review by the expert of the systemic lupus erythematosus subjects, the expert proposed other criteria to determine the diagnosis and its confirmation. The expert reviewed all remaining subjects and applied these criteria.

### Statistical analysis

#### Main analyses

The main analysis included all confirmed AD cases with a known date of first symptom within the follow-up period (i.e., the date of first symptom was set as the date of disease occurrence). A known date of first symptom was either the date (from the free text) that a symptom was said by the patient to have started, or, if this was not available, the date the first symptom was reported in CPRD. If the date of first symptom was within the one year follow-up period but the date of diagnosis was after this period then this subject was not included as a case. The incidence rates of AD during the one year follow-up period were calculated as the number of cases divided by the total person-time. The individual person-time was defined as the time between the study start date and the end of follow-up period (one year from study start date), subject's date of death, CPRD de-enrolment date, date of unspecified HPV vaccine or 4vHPV, or date of first symptom of AD, whichever occurred first. The comparison of the incidence rates of AD (co-primary endpoints and individual diseases with more than 10 cases in the female cohorts: these concerned Crohn's disease, autoimmune thyroiditis, and diabetes mellitus type 1) was done using a Poisson regression model, with number of events as dependent variable, exposure status as independent variable, and age as covariate, and the log of person-time as an offset. The IRR (females: exposed/historical, males: concurrent/historical) was derived as the exponential of the coefficient associated with the exposure status and its 95% Wald CI. A Poisson regression model adjusted for time effect was performed for the AD for which a statistically significant difference in incidence rates was observed between the 2 male cohorts. This model included the 4 cohorts and a specific contrast for estimating the difference between the 2 female cohorts adjusted for the difference between the 2 male cohorts.

#### Sensitivity analyses

The following sensitivity analyses were performed:

Analyses of all cases (confirmed and non-confirmed) with known date of first symptom within the follow-up period;

Analyses using cases with a known or imputed date of first symptom (confirmed cases only, and confirmed and non-confirmed cases combined) within the follow-up period. In case of missing date of first symptom, a date was imputed using the disease-specific median number of days between the date of diagnosis and the known date of first symptom of all confirmed and non-confirmed cases. If the (imputed) date of first symptom was within the defined risk period but the date of diagnosis was after the risk period then this subject was not included as a case;

Analyses where the date of diagnosis was set as the date of disease occurrence (confirmed cases only, and confirmed and non-confirmed cases combined);

Analyses using, in addition to age, also region, other vaccination, and healthcare resource utilization during the year prior to the study start date as covariates.

### Self-controlled case-series

A SCCS analysis for both co-primary endpoints and individual diseases was performed for the exposed female cohort. For the main SCCS, the risk period was one year after the first AS04-HPV-16/18 vaccine dose; a buffer period was defined as the 6 months after the end of the risk period and the control period was defined as one year after the end of the buffer period.

Potential pre-existing autoimmune conditions may influence vaccination status. For this reason, the control period did not include a pre-vaccine period. The relative incidence rate was calculated for the coprimary endpoints and individual diseases between risk and control periods as the ratio of the incidence rate in the risk period versus the incidence rate in the control period. Confirmed AD cases with a date of first symptom within these 30 months were included in the SCCS analysis. If the date of first symptom was within the defined risk period but the date of diagnosis was after the risk period then the case was excluded from the SCCS analysis. The same rule was applied for cases occurring in the control period because no diagnosis that occurred after the end of the control period was included in the study. The reason for the use of this rule was to avoid a bias in the number of cases occurring in the risk period.

### Post-hoc analyses

Post-hoc analyses included a descriptive analysis of time-to-onset of all confirmed autoimmune thyroiditis cases. Moreover, an additional subject profile review was performed for all confirmed and non-confirmed autoimmune thyroiditis cases with a known date of first symptom within the follow-up period in order to classify the cases as hypo- or hyperthyroiditis and to derive IRR's for these subtypes separately. Lastly, an analysis excluding subjects from Northern Ireland, Scotland, and Wales was performed, because a large proportion of confirmed autoimmune thyroiditis cases were observed in this region (10.2%), while this region represented less than 0.1% of the overall study population cohorts.

### Sample size

For the cohort design, by hypothesizing that the incidence rates of neuro-inflammatory AD vary between 1 and 10/100,000 person-years and the incidence rates of other AD vary between 50 and 100/100,000 person-years, cohorts of 50,000 subjects each should allow the detection, with 80% power, of a relative risk between 18.7 and 3.7 and between 2.0 and 1.6 respectively for the neuro-inflammatory AD and other AD (our 2 co-primary endpoints). Because of risk of loss to follow-up and missing data, the sample size was increased by 30% for a total of 65,000 subjects in each cohort.

## Supplementary Material

KHVI_A_1199308_Supplement.docx

## Abbreviations

AD: autoimmune diseases
CI: confidence interval
CPRD GOLD: Clinical Practice Research Datalink General Practice OnLine Database
GP: general practices
GSK: GlaxoSmithKline
HES: Hospital Episode Statistics
HPV: human papillomavirus
IRR: incidence rate ratio
MHRA: Medicines and Healthcare products Regulatory Agency
RTI: Research Triangle Institute
SCCS: self-controlled case-series
UK: United Kingdom
US: United States
VIVIANE: Human Papilloma **Vi**rus: **V**accine Immunogenicity and Efficacy
