# Supplementary material for: Risk of new onset autoimmune disease in 9- to 25-year-old women exposed to human papillomavirus-16/18 AS04-adjuvanted vaccine in the United Kingdom
Source: Hum Vaccin Immunother. 2016 Jul 18;12(11):2862–71. doi: 10.1080/21645515.2016.1199308 (PMC5137515; doi:10.1080/21645515.2016.1199308)
Supplement: KHVI_A_1199308_Supplement.docx [file khvi-12-11-1199308-s001.docx]

**Supplemental Tables**

Table S1: Incidence rate per 100,000 person-years and incidence rate ratios* of co-primary endpoints and individual diseases with >10 cases in female cohorts – sensitivity analysis with imputed date of first symptom

|  |  | **AS04-HPV-16/18 vaccine exposure (total PY=64,730)** | | **Unexposed historical female cohort**  **(female total PY=64,844)** | | |  |
| --- | --- | --- | --- | --- | --- | --- | --- |
| **Diseases** |  | **n** | **IR per 100,000 PY (95% CI)** | **n** | | **IR per 100,000 PY (95% CI)** | **IRR* (95% CI) EXP/NNEXP** |
| **Co-primary endpoints** | | | | | | |  |
| Neuroinflammatory/ ophthalmic AD | Confirmed cases | 0 | 0.00 (0.00; 5.70) | 1 | 1.54 (0.04; 8.59) | | 1.00 (0.06; 16.08) |
|  | All cases | 5 | 7.72 (2.51; 18.03) | 9 | 13.88 (6.35; 26.35) | | 0.56 (0.19; 1.66) |
| Other AD | Confirmed cases | 42 | 64.89 (46.76;87.71) | 33 | 50.89 (35.03;71.47) | | 1.27 (0.81; 2.01) |
|  | All cases | 60 | 92.69 (70.73; 119.31) | 52 | 80.19 (59.89; 105.16) | | 1.16 (0.79; 1.67) |
| **Individual diseases with >10 cases in female cohorts** | | | |  |  | |  |
| Autoimmune thyroiditis | Confirmed cases | 16 | 24.72 (14.13; 40.14) | 8 | 12.34 (5.33; 24.31) | | 2.00 (0.86; 4.67) |
|  | All cases | 32 | 49.44 (33.81; 69.79) | 27 | 41.64 (27.44; 60.58) | | 1.19 (0.71; 1.98) |
| Crohn’s disease | Confirmed cases | 7 | 10.81 (4.35; 22.28) | 5 | 7.71 (2.50; 17.99) | | 1.41 (0.45; 4.43) |
|  | All cases | 9 | 13.90 (6.36; 26.39) | 5 | 7.71 (2.50; 17.99) | | 1.81 (0.61; 5.39) |
| Type 1 diabetes mellitus | Confirmed cases | 10 | 15.45 (7.41; 28.41) | 18 | 27.76 (16.45; 43.87) | | 0.56 (0.26; 1.21) |
|  | All cases | 10 | 15.45 (7.41; 28.41) | 18 | 27.76 (16.45; 43.87) | | 0.56 (0.26; 1.21) |
|  |  |  |  |  |  | |  |
|  |  | **Unexposed concurrent male cohort**  **(total PY=64,865)** | | **Unexposed historical male cohort**  **(total PY=64,874)** | | |  |
| **Diseases** |  | **n** | **IR per 100,000 PY (95% CI)** | **n** | | **IR per 100,000 PY (95% CI)** | **IRR* (95% CI) MALE/HIST** |
| **Co-primary endpoints** | | | | | | |  |
| Neuroinflammatory/ ophthalmic AD | Confirmed cases | 2 | 3.08 (0.37; 11.14) | 1 | | 1.54 (0.04; 8.59) | 1.89 (0.17; 20.94) |
|  | All cases | 7 | 10.79 (4.34; 22.24) | 4 | | 6.17 (1.68; 15.79) | 1.82 (0.53; 6.24) |
| Other AD | Confirmed cases | 33 | 50.88 (35.02; 71.45) | 19 | | 29.29 (17.63; 45.74) | 1.78 (1.01; 3.14) |
|  | All cases | 38 | 58.58 (41.46; 80.41) | 29 | | 44.70 (29.94;64.20) | 1.35 (0.83; 2.19) |
| **Individual diseases with >10 cases in female cohorts** | | | |  | |  |  |
| Autoimmune thyroiditis | Confirmed cases | 1 | 1.54 (0.04; 8.69) | 0 | | 0.00 (0.00; 5.69) | Not done |
|  | All cases | 5 | 7.71 (2.50; 17.99) | 7 | | 10.79 (4.34; 22.23) | 0.73 (0.23; 2.31) |
| Crohn’s disease | Confirmed cases | 5 | 7.71 (2.50; 17.99) | 1 | | 1.54 (0.04; 8.59) | 5.19 (0.60; 44.68) |
|  | All cases | 5 | 7.71 (2.50; 17.99) | 2 | | 3.08 (0.37; 11.14) | 2.55 (0.49; 13.23) |
| Type 1 diabetes mellitus | Confirmed cases | 23 | 35.46 (22.48; 53.21) | 12 | | 18.50 (9.56; 32.31) | 1.89 (0.94; 3.82) |
|  | All cases | 23 | 35.46 (22.48; 53.21) | 14 | | 21.58 (11.80; 36.21) | 1.65 (0.85; 3.20) |

AD = autoimmune disease; CI = confidence interval; EXP = exposed female cohort; HIST = unexposed historical male cohort; IRR = incidence rate ratio; MALE = unexposed concurrent male cohort; n = number of subjects; NNEXP = unexposed historical female cohort; PY = person-years
* Adjusted for age group (9-17 years, 18-25 years)

Table S2: Incidence rate per 100,000 person-years and incidence rate ratios* of co-primary endpoints and individual diseases with >10 cases in female cohorts – sensitivity analysis with date of diagnosis as date of disease onset

|  |  | **AS04-HPV-16/18 vaccine exposure**  **(total PY=64,852)** | | **Unexposed historical female cohort**  **(total PY=64,893)** | | |  |
| --- | --- | --- | --- | --- | --- | --- | --- |
| **Diseases** |  | **n** | **IR per 100,000 PY (95% CI)** | **n** | | **IR per 100,000 PY (95% CI)** | **IRR* (95% CI) EXP/NNEXP** |
| **Co-primary endpoints** | | | | | | |  |
| Neuroinflammatory/ ophthalmic AD | Confirmed cases | 1 | 1.54 (0.04; 8.59) | 1 | 1.54 (0.04; 8.59) | | 1.00 (0.6; 16.10) |
|  | All cases | 6 | 9.25 (3.40; 20.14) | 10 | 15.41 (7.39; 28.34) | | 0.60 (0.21; 1.65) |
| Other AD | Confirmed cases | 58 | 89.44 (67.91; 115.62) | 52 | 80.13 (59.85; 105.08) | | 1.12 (0.77; 1.62) |
|  | All cases | 87 | 134.15 (107.45; 165.48) | 85 | 130.99 (104.63; 161.97) | | 1.024 (0.76; 1.38) |
| **Individual diseases with >10 cases in female cohorts** | | | |  |  | |  |
| Autoimmune thyroiditis | Confirmed cases | 23 | 35.47 (22.48; 53.22) | 15 | 23.12 (12.94; 38.13) | | 1.53 (0.80; 2.94) |
|  | All cases | 48 | 74.01 (54.57; 98.13) | 46 | 70.89 (51.90; 94.55) | | 1.04 (0.69; 1.56) |
| Crohn’s disease | Confirmed cases | 11 | 16.96 (8.47; 30.35) | 9 | 13.87 (6.34; 26.33) | | 1.23 (0.51; 2.96) |
|  | All cases | 13 | 20.05 (10.67; 34.28) | 9 | 13.87 (6.34; 26.33) | | 1.45 (0.62; 3.39) |
| Type 1 diabetes mellitus | Confirmed cases | 11 | 16.96 (8.47; 30.35) | 20 | 30.82 (18.83; 47.60) | | 0.55 (0.26; 1.15) |
|  | All cases | 11 | 16.96 (8.47; 30.35) | 20 | 30.82 (18.83; 47.60) | | 0.55 (0.26; 1.15) |
|  |  |  |  |  |  | |  |
|  |  | **Unexposed concurrent male cohort**  **(total PY=64,897)** | | **Unexposed historical male cohort**  **(total PY=64,891)** | | |  |
| **Diseases** |  | **n** | **IR per 100,000 PY (95% CI)** | **n** | | **IR per 100,000 PY (95% CI)** | **IRR* (95% CI) MALE/HIST** |
| **Co-primary endpoints** | | | | | | |  |
| Neuroinflammatory/ ophthalmic AD | Confirmed cases | 2 | 3.08 (0.37; 11.13) | 1 | | 1.54 (0.04; 8.59) | 1.89 (0.17; 20.94) |
|  | All cases | 9 | 13.87 (6.34; 26.33) | 3 | | 4.62 (0.95; 13.51) | 3.11 (0.84; 11.52) |
| Other AD | Confirmed cases | 45 | 69.34 (50.58; 92.78) | 33 | | 50.85 (35.01; 71.42) | 1.39 (0.88; 2.18) |
|  | All cases | 56 | 86.29 (65.18; 112.06) | 48 | | 73.97 (54.54; 98.07) | 1.19 (0.81; 1.75) |
| **Individual diseases with >10 cases in female cohorts** | | | |  | |  |  |
| Autoimmune thyroiditis | Confirmed cases | 2 | 3.08 (0.37; 11.13) | 0 | | 0.00 (0.00; 5.69) | Not done |
|  | All cases | 10 | 15.41 (7.39; 28.34) | 8 | | 12.33 (5.32; 24.29) | 1.25 (0.49; 3.18) |
| Crohn’s disease | Confirmed cases | 15 | 23.11 (12.94; 38.12) | 8 | | 12.33 (5.32; 24.29) | 1.94 (0.82; 4.59) |
|  | All cases | 16 | 24.65 (14.09; 40.04) | 10 | | 15.41 (7.39; 28.34) | 1.64 (0.74; 3.62) |
| Type 1 diabetes mellitus | Confirmed cases | 23 | 35.44 (22.47; 53.18) | 12 | | 18.49 (9.56; 32.30) | 1.89 (0.94; 3.82) |
|  | All cases | 23 | 35.44 (22.47; 53.18) | 14 | | 21.58 (11.80; 36.20) | 1.65 (0.85; 3.20) |

AD = autoimmune disease; CI = confidence interval; EXP = exposed female cohort; HIST = unexposed historical male cohort; IRR = incidence rate ratio; MALE = unexposed concurrent male cohort; n = number of subjects; NNEXP = unexposed historical female cohort; PY = person-years
* Adjusted for age group (9-17 years, 18-25 years)

Table S3: Relative incidence between risk and control periods for confirmed cases (self-controlled case-series analysis)

|  | **Risk period (n)** | **Control period (n)** | **Relative incidence** |
| --- | --- | --- | --- |
| **Diseases** |  |  | **(95% CI)** |
| **Co-primary endpoints** | | | |
| Neuroinflammatory/ophthalmic AD | 0 | 2 | 0.00 (0.00- ) |
| Other AD | 38 | 28 | 1.36 (0.83-2.21) |
| **Individual diseases with >10 cases in risk and control period** | | | |
| Autoimmune thyroiditis | 15 | 11 | 1.36 (0.63-2.97) |
| Type 1 diabetes mellitus | 8 | 7 | 1.14 (0.41-3.15) |

AD = autoimmune disease; CI = confidence interval; n = number of subjects;

Table S4: Algorithm for GBS case identification in CPRD and/or HES

| **Guillain Barré Syndrome** | Guillain Barré Syndrome (GBS) cases will be identified where:   - in *Clinical and Referral file*: a medcode for GBS is listed (see eTable 2) - in *HES (HES_diagnosis_epi file)*: an ICD10 diagnosis code for GBS is listed (see eTable 2)   Eventdate should be between reference date and (reference date+365 days) for unexposed cohort and between reference date and (reference date+30months) for exposed cohort.  Freetext related to GBS in the study period will be retrieved and the case will be sent for expert review.  Additional information will be retrieved in order to complete the patient profile, the eventdate should be 1 year before the reference date or during the follow-up period:   - in *Therapy file*: all prodcodes - in *Test file*: Nerve conduction studies (enttype=343) and cerebrospinal fluid examination (enttype=410 ) |
| --- | --- |

Table S5: Medical codes and ICD-10 codes for Guillain Barre Syndrome:

| **Medcode (CPRD-GOLD Medical Code Events)** | **Read Code** | **Read Description** | **ICD-10 codes** | **Review** |
| --- | --- | --- | --- | --- |
| 28294 | F326100 | Polyneuritis cranialis | G52.7 | Possible |
| 44512 | F364.00 | Idiopathic progressive polyneuropathy | G60.3 | Possible |
| 14884 | F36y.00 | Other idiopathic peripheral neuropathy | G60 | Possible |
| 1607 | F370000 | Guillain-Barre syndrome | G61.0 | Y |
| 24216 | F370100 | Postinfectious polyneuritis | G61.0 | Possible |
| 33841 | F370200 | Miller-Fisher syndrome | G61.0 | Y |
| 63555 | F374z00 | Polyneuropathy in disease NOS | G63 | Possible |
| 31551 | F37X.00 | Inflammatory polyneuropathy, unspecified | G61.9 | Possible |
| 69047 | F37y000 | Serum neuropathy | G61.1 | Possible |
| 96256 | F37y100 | Axonal sensorimotor neuropathy | G60 | Y |
| 15481 | F37z.00 | Toxic or inflammatory neuropathy NOS | G61.9 | Possible |
| 24226 | F37z.11 | Polyneuropathy unspecified | G62.9 | Possible |
| 55076 | Fyu7.00 | [X]Polyneuropathies & other disord of peripheral nerv syst | G60/G64 | Possible |
| 97449 | Fyu7000 | [X]Other hereditary and idiopathic neuropathies | G60.8 | Possible |
| 97306 | Fyu7200 | [X]Other specified polyneuropathies | G62.8 | Possible |

Table S6: Variables directly extracted from CPRD GOLD

|  | **Column name** | **Field name** | **Description** | **CPRD GOLD file** |
| --- | --- | --- | --- | --- |
| ***1*** | ***Patient Identifier*** | ***patid*** | Unique identifier given to a patient | patient |
| ***2*** | ***Patient Gender*** | ***gender*** | Patient’s gender | patient |
| ***3*** | ***Birth Month*** | ***mob*** | Patient’s month of birth (for those aged under 16) | patient |
| ***4*** | ***Birth Year*** | ***yob*** | Patient’s year of birth | patient |
| ***5*** | ***Practice Identifier*** | ***pracid*** | Unique identifier given to a specific practice | practice |
| ***6*** | ***Practice Region*** | ***region*** | Practice region: Value to indicate where in the United Kingdom the practice is based | practice |
| ***7*** | ***Death Date*** | ***deathdate*** | Date of death of patient – derived using an algorithm | patient |
| ***8*** | ***First Registration Date*** | ***frd*** | First registration date: Date the patient first registered with the practice. If patient only has ‘temporary’ records, the date is the first encounter with the practice; if patient has ‘permanent’ records it is the date of the first ‘permanent’ record (excluding preceding temporary records) | patient |
| ***9*** | ***Current Registration Date*** | ***crd*** | Date the patient’s current period of registration with the practice began (date of the first ‘permanent’ record after the latest transferred out period). If there are no ‘transferred out periods’, the date is equal to ‘frd’ | patient |
| ***10*** | ***Registration Gaps*** | ***reggap*** | Number of days missing in the patients registration details | patient |
| ***11*** | ***Registration Status*** | ***regstat*** | Registration status: Status of registration detailing gaps and temporary patients | patient |
| ***12*** | ***Transfer Out Date*** | ***tod*** | Date the patient transferred out of the practice | patient |
| ***13*** | ***Transfer Out Reason*** | ***toreason*** | Reason the patient transferred out of the practice. Includes 'Death' as an option | patient |
| ***14*** | ***Up To Standard Date*** | ***uts*** | Date at which the practice data is deemed to be of research quality. Derived using an algorithm that primarily looks at practice death recording and gaps in the data | practice |
| ***15*** | ***Acceptable Patient Flag*** | ***accept*** | Flag to indicate whether the patient has met certain quality standards: 1 = acceptable, 0 = unacceptable | practice |
| ***16*** | ***Matching CPRD-HES*** | ***HES_e*** | Flag (0,1) indicating whether patient is eligible for linkage to HES data | linkage_eligibility |

^1^ **dd/mm/yyyy**: Valid dates are in the format DD/MM/YYYY. Missing dates are NULL, and invalid dates are set to 01/01/2500.

^2^ **PAT_GAP:** Number of days between patient’s transferred out date and re-registration date for the patient’s ‘transferred out periods’, regardless of whether the transfer was internal or not.

^3^ **PAT_STAT:** Transferred out period is the time between a patient transferring out and re-registering at the same practice. If the patient has transferred out for a period of more than 1 day, and the transfer is not internal, this value is incremented. 0 means continuous registration, 1 means one ‘transferred out period’, 2 means two periods, etc. If the patient only has ‘temporary’ records then this value is set to 99.

Table S7: Variables derived from CPRD GOLD

|  | **Column Name** | **Algorithms** |
| --- | --- | --- |
| ***17*** | ***Date of Cervarix vaccination*** | Search for the subject in *Immunisation file* where:   - Immstype equals 67 (HPVCER) and status equals 1 - Retrieve the eventdates   For the subjects with at least one recorded dose in *Immunisation file*:  1) Search for additional Cervarix vaccination in *Therapy file* where:   - Cervarix prodcode = 36952   The vaccinations from *Therapy* file will be considered as additional Cervarix vaccination if the eventdate is not equal to eventdate (+/- 14 days) from *Immunisation file*.  2) Search for additional Cervarix vaccination in *Clinical file* (medcode=93489 93621 95554):  - if the eventdate is equal to eventdate from *Immunisation or Therapy file* then vaccination is similar than the one from *Immunisation or Therapy* *file*.  - if the eventdate is not equal to eventdate but in an interval of +/- 14 days from *Immunisation or Therapy file* then the vaccination is not an additional unspecified HPV vaccination –  ntPV. the date is different, the dose is considered as an unspecified additional doses  - if the eventdate is not equal to eventdate +/- 14 days from *Immunisation or Therapy file* then the vaccination is an additional unspecified HPV vaccination  If the 1st dose of Cervarix is between 01Sep2008 and 31Dec2010, the subject will be included in the exposed cohort. The date of 1st dose of Cervarix vaccination is the study start date for exposed cohort.  Eventdate of all of recorded Cervarix doses will be retrieved. |
| ***18*** | ***Date of unspecified HPV or Gardasil vaccine*** | This variable will retrieve a date of unspecified HPV OR Gardasil vaccination.  Search for the subject in *Immunisation file* where:   - Medcode in (93489, 93621, 95554) (HPV 1st, 2d, 3rd dose) AND immstype equals 58 or not specified - Retrieve the eventdates   Search for additional unspecified HPV or Gardasil vaccine in *Therapy file* where:   - prodcode = 32424 37955 /Gardasil prodcode =32147   The vaccinations from *Therapy file* will be considered as additional unspecified HPV or Gardasil vaccination if the eventdate is not equals to eventdate (+/- 14 days) from *Immunisation file*.  Search for additional HPV vaccinations in *Clinical file* (medcode=93489 93621 95554):  - if the eventdate is equal to eventdate from *Immunisation or Therapy file* then vaccination is similar than the one from *Immunisation or Therapy file*.  - if the eventdate is not equal to eventdate but in an interval of +/- 14 days from *Immunisation or Therapy file* then the vaccination is not an additional unspecified HPV vaccination –  ntPV. the date is different, the dose is considered as an unspecified additional doses.  - if the eventdate is not equal to eventdate +/- 14 days from *Immunisation or Therapy file* then the vaccination is an additional unspecified HPV vaccination |
| ***19*** | ***Date of any other vaccine*** | This variable checks if a vaccine (other than HPV) was administered during the year before the study start date.  Search for the subject in *Immunisation file and Therapy file* if a medcode (for vaccine) exist, retrieve eventdate, immstype and medcode and status=1 for *Immunisation file*. Search in *Therapy file* if a prodcode for vaccine exist, retrieve eventdate, drugsubstance, productname.  Eventdate of vaccination should be between the study start date -365 and end of follow-up. |
| ***20*** | ***Date of birth*** | Date of birth will be derived from month of birth (mob) and year of birth (yob) in *Patient file*.  If month of birth and year of birth are present, the date of birth will be read as “15mmyyyy”. If month of year is not present, it will be read as “30JUNyyyy”. |
| ***21*** | ***CPRD Start Date*** | From *Patient and Practice file*:  If crd < Up to Standard Date then CPRD Start Date= Up to Standard Date (uts)  If crd > Up To Standard Date then CPRD Start Date=Current registration Date (crd) |
| ***22*** | ***Health care resource utilization*** | The number of GP/primary care consultations during the year before the study start date will be retrieved from *Consultation file*. |
| ***23*** | ***Date of autoimmune disease diagnosis*** | The autoimmune diagnosis will be identified by applying the algorithm 24 from 42. If the same recorded medcode has more than one event date, then the first event will be used as the first date of autoimmune diagnosis. |
| ***24-42*** | ***Autoimmune disease name*** | Each autoimmune disease will be retrieved from algorithms (available upon request) |
